# Editorial

**Published:** 1841-01-02

**Authors:** 


THE MEDICAL EXAMINER.

PHILADELPHIA, JANUARY 2, 1841.

### OLD REMEDIES—AGARIC.

In many of the cases which we republish from the European periodicals, our readers must be struck with the frequent employment of *old* remedies, which, with us, are almost unknown,—remedies, however, deserving of esteem from their very antiquity; for the continued experience, in some instances of centuries, has only served to confirm their worth.

We fear that the proverbial fondness of our countrymen for novelty, and their happy disposition to improve upon the labours of their predecessors, may lead them occasionally to reject, as obsolete, materials of great value. The disposition is one we would foster; for to it alone will medical *science* owe its progressive improvement; but medical *art* must be amended by a different process. The latter will always remain, to a considerable extent, under the control of a rational empiricism. The action of remedies can rarely be inferred. Experiment only can determine it in the first instance, and experience is afterwards required to confirm it. The combined testimony of the two once obtained, we have a new *fact* added to our store, which it is unwise at a future period heedlessly to cast away. The operation is not liable to vary much with date; and when we find an accumulation of consistent evidence demonstrative of the effects of an agent a cenury since, we may infer that identical effects, will be produced by its employment now. Yet, in divesting the materia chirurgica of a mass of rude and barbarous material, much that had been thus stamped as valuable by the seal of time has certainly been sacrificed, and we fancy it will not be unprofitable to rescue from oblivion some of these antiquated remedies, and to test their present usefulness.

The different species of Agaric, for example, particularly the *Boletus Ignarius*, enjoyed a continually increasing reputation as a styptic, from the middle of the sixteenth to the middle of the eighteenth century, and at this latter period, its efficacy in arresting hæmorrhage, even from large arteries, was so generally admitted, that in France an animated discussion took place in reference to the question, whether its advantages were not superior in many instances to those obtained from the employment of a ligature. In England and Holland also it had its advocates. Mr. Warner (of Guy's Hospital, London,) attempted to show that after amputation of the leg, the largest trunks were so rapidly and completely obliterated, that a mere temporary application of the agaric was all that was required. In five cases of amputation of the leg, no secondary hæmorrhage occurred; in one instance a slight discharge of blood was observed *one hour* after the operation, requiring the removal of the dressings, when it was seen to proceed from a small vessel, which had not been protected by the styptic; he embraced the opportunity of removing the agaric carefully from the larger trunks, which he found so completely closed, that with a loosened tourniquet not a drop of blood escaped. Now, although there may be exaggeration in these statements, and the compression by which the agaric was supported may have had its share in arresting the hæmorrhage, still they prove beyond a doubt the really *hemostatic* qualities of the remedy; and the possession of more certain means to attain the same end is not motive sufficient to authorise its entire rejection. The ligature and torsion cannot exclusively be relied upon; in operations opening bony cavities, as the removal of tumours from the antrum, a necrosed sequestrum from a new shell, in wounds involving an erectile tissue, &c., the flow of blood is frequently profuse, and yet the ligature and torsion may be inapplicable or counter-indicated, and cauterisation, or the much milder application of styptics are our only resource.

How important then is it to retain one of this class, which, without being destructive of tissue, will be efficient as a hæmostatic. The agaric is now generally considered as a simple absorbent, but this, we are convinced, is an unjust limitation of its powers. Morand ingeniously referred its styptic action to its absorbent qualities, on the supposition, that by the imbibition of the serous portion of the blood, the agaric swelled and produced a more methodical compression; while at the same time, the removal of the fluid particles rendered the fibrine more disposed to coagulate, the conjoined action being required to arrest the hæmorrhage. But the agaric, *in its natural state*, appears neither to absorb or swell, while its styptic action is in some cases instantaneous.

In an operation which we witnessed within a week, requiring the removal of a portion of the scalp, the arterial hæmorrhage was very free; and one of the larger branches of the left temporal pulsated visibly on the cut surface, which was more than an inch square. A piece of agaric capable of covering two-thirds of the wound, was lightly placed upon the left portion, and the surface covered instantly ceased to bleed. A second piece was placed on the right side, and the effect was the same. A small interval existed between the two upon which a third piece was permitted to rest for a few seconds, and upon its removal the florid blood was found converted into a black coagulum, and not a single drop subsequently escaped. After forty-eight hours the dressings were removed, a healthy granular surface was exhibited, and the agaric was apparently as dry as at the moment of application.

